# The significance of heart rate variability in patients with frequent premature ventricular complex originating from the ventricular outflow tract

**DOI:** 10.1002/clc.24174

**Published:** 2023-10-19

**Authors:** Baowei Zhang, Jinbo Yu, Yizhang Wu, Xiaorong Li, Xin Xie, Aibin Tao, Bing Yang

**Affiliations:** ^1^ Center of Cardiology, Shanghai East Hospital Tongji University School of Medicine Shanghai China; ^2^ Department of Cardiology the affiliated People's Hospital of Jiangsu University Zhenjiang Jiangsu China

**Keywords:** autonomic nervous system, closely, heart rate variability, premature ventricular complex, ventricular outflow tract

## Abstract

**Background:**

As an indicator of cardiac autonomic nervous activity, heart rate variability (HRV) is closely linked to premature ventricular complexes (PVCs). However, its role in patients with frequent PVCs originating from the ventricular outflow tract remains unclear.

**Hypothesis:**

Here, we hypothesize that there may be alterations in HRV among patients with frequent PVCs originating from the ventricular outflow tract, which could play significant roles in the management of such patients.

**Methods:**

A retrospective study was conducted, including 106 patients with frequent outflow tract PVCs and 106 healthy participants as controls. HRV was assessed based on the 24‐hour Holter recording. The originating foci of PVCs were identified during radiofrequency catheter ablation.

**Results:**

Patients with frequent outflow tract PVCs exhibited decreased levels of high frequency (HF), standard deviation of all NN intervals, and standard deviation of the average NN intervals, but increased ratios of low frequency to HF (LF/HF ratio), even after propensity score‐matched analysis. Further investigation revealed that patients with PVCs originating from right ventricular outflow tract (RVOT) had much higher LF/HF ratios. Multivariate logistic regression analysis demonstrated that the LF/HF ratio was independently associated with PVCs originating from RVOT. Receiver operating characteristics curve indicated that the LF/HF ratio effectively determined the origin of PVCs (the area under the curve = 0.75, *p* < .001).

**Conclusions:**

Patients with frequent outflow tract PVCs exhibited impaired HRV. Additionally, the LF/HF ratio played a significant role in determining the origin of outflow tract PVCs.

## Graphical abstract

A total of 106 frequent outflow tract premature ventricular complexes (OT‐PVCs) patients undergoing radiofrequency catheter ablation and 106 healthy participants were included in the study to examine the significance of heart rate variability (HRV) in patients with frequent OT‐PVCs. Patients with OT‐PVCs showed impaired HRV, while those with RVOT‐PVCs exhibited higher low frequency to high frequency ratios compared to patients with LVOT‐PVCs.

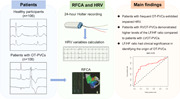

## 1 INTRODUCTION

The cardiac autonomic nervous system (ANS), comprising the sympathetic and parasympathetic nervous systems, plays a crucial role in modulating ventricular arrhythmias (VAs).^1^ In VAs associated with structural heart disease, the activation of sympathetic nervous system may provide both the trigger and substrate for the initiation and maintenance of VAs.^2^ Studies have demonstrated that inhibition of the sympathetic nervous system and stimulation of the parasympathetic nervous system can reduce the occurrence of VAs in patients with ischemic heart diseases.^3^ However, the impact of the ANS on idiopathic VAs appears to be more intricate, as both increased sympathetic and parasympathetic nervous system activity have been shown to correlate with higher VAs burdens.^4^, ^5^

Idiopathic outflow tract VAs (OT‐VAs), characterized by electrocardiographic patterns consistent with left or right bundle brunch block and inferior axis morphology, account for approximately two‐thirds of idiopathic VAs.^6^ While the right ventricular outflow tract (RVOT) is commonly recognized as the primary site of origin for OT‐VAs, up to one‐third of OT‐VAs are believed to originate from the left ventricular outflow tract (LVOT).^7^ In clinical practice and electrophysiology laboratories, OT‐VAs are frequently triggered by physical exercise, stress, and catecholamine infusion, while they can be attenuated by β‐blockers or Valsalva maneuvers. These observations suggest that the cardiac ANS may play a significant role in the initiation and maintenance of OT‐VAs.^8^ Heart rate variability (HRV), derived from the oscillation between consecutive heartbeats, is the most common indirect measure of cardiac ANS function.^9^ Previous studies have shown that the initiation of RVOT‐VAs was associated with sympathetic system activation, as assessed by HRV analysis.^10^, ^11^ However, the utility of HRV as a straightforward approach for evaluating cardiac autonomic function in patients with OT‐VAs remains uncertain. Additionally, the potential discrepancy in HRV between patients with RVOT‐VAs and LVOT‐VAs is still unclear. The purpose of this study was to examine the significance of HRV in patients with RVOT‐VAs and LVOT‐VAs.

## 2 MATERIALS AND METHODS

### 2.1 Patients

To investigate the cardiac ANS function in patients with frequent idiopathic outflow tract premature ventricular complexes (OT‐PVCs), a retrospective study was conducted in Zhenjiang First People's Hospital and Shanghai East hospital from December 2014 to July 2020. The study included 133 patients who underwent radiofrequency catheter ablation (RFCA) for their intolerant and drug‐resistant symptoms caused by frequent PVCs. These patients had either PVC counts > 10 000 beats/24 h or PVC burden > 10% as assessed by Holter monitoring. The study protocol adhered to the Declaration of Helsinki and received approval from the institutional ethics committee board of Zhenjiang First People's Hospital. Individual consent for this retrospective analysis was waived.

The diagnosis of idiopathic OT‐PVC was based on electrocardiography (ECG) findings, including left or right bundle branch block and inferior axis morphology, without any evidence of structural heart disease on echocardiography. Based on the originating foci of PVCs identified during RFCA, patients with frequent OT‐PVCs were categorized into two groups: RVOT group and LVOT group. Exclusion criteria consisted of the following: (1) unsuccessful ablation or unclarified origin of PVCs (*n* = 13); (2) evident structural heart disease or heart failure (*n* = 5); (3) long‐term poor glycemic control or evidence of diabetic neuropathy in diabetics (*n* = 1); (4) severe renal dysfunction, liver dysfunction, thyroid disorder, or malignancy (*n* = 4); (5) nervous system disorders (*n* = 1); (6) presence of other identified arrhythmias (*n* = 3). Additionally, a control group of 106 healthy participants was included (Supporting Information: Figure S1).

### 2.2 Data collection and HRV analyses

All patients underwent evaluation of elementary clinical characteristics, echocardiography, and Holter recording. PVCs were classified into three distribution patterns based on the relationship between hourly PVC counts and the corresponding average heart rate during Holter monitoring (Supporting Information: Figure S2A).

HRV was computed utilizing a validated three‐channel device from the 24‐hour Holter recording (Seer Light Dynamic Electrocardiogram record system; GE Healthcare). During the Holter examination, all participants were instructed to maintain their normal activities. All HRV parameters were calculated after removing ectopic beats. Frequency domain analysis and time domain analysis are the two most common methods used to evaluate HRV on Holter recordings. Frequency domain analysis is based on spectral analysis of a sequence of RR intervals and provides information about the distribution of power across different frequency ranges. The variables obtained from frequency domain analysis include the high frequency (HF) component (0.15−0.4 Hz), low frequency (LF) component (0.04−0.15 Hz), and very LF (VLF) component (0.003−0.04 Hz). Additionally, the LF to HF (LF/HF) ratio is automatically calculated by the software. The VLF and LF components of HRV have a more complex physiology that involves both sympathetic and parasympathetic activities, while the HF component represents parasympathetic tone. The LF/HF ratio serves as an index of the balance between sympathetic and parasympathetic activity in the cardiac ANS.

On the other hand, time domain measures are calculated using statistical and mathematical analysis of RR intervals. Commonly used variables in time domain analysis include the standard deviation of all NN intervals (SDNN), the standard deviation of the average NN intervals (SDANN), the square root of the mean of the squares of successive differences between adjacent NN intervals (rMSSD), and the percentage of normal RR intervals that differ by more than 50 millisecond (pNN50). Both SDNN and SDANN reflect a greater contribution of sympathetic nervous activity to HRV, and decreased levels of SDNN and SDANN indicate increased sympathetic tone. rMSSD and pNN50 are reliable indicators of parasympathetic tone.^12^, ^13^, ^14^

### 2.3 Electrophysiological study and ablation

Electrophysiological studies were conducted in all patients with frequent OT‐PVCs in fasting state after informed consent for the procedure had been obtained. All anti‐arrhythmia drugs, except amiodarone, were discontinued for a minimum of 5 half‐lives. A 4‐mm‐tip nonirrigated catheter (Navistar; Biosense Webster) or an irrigation catheter (Navistar ThermoCool; Biosense Webster) was utilized for mapping and ablation. The originating foci of PVCs were identified by activation mapping and pace mapping under the guidance of CARTO3 system (Biosense Webster) or fluoroscopy. Briefly, activation mapping is guided by the bipolar electrogram, and the earliest bipolar activation is used to identify the origin of OT‐PVCs. Additionally, in cases where the earliest activation site displayed a QS pattern on unipolar recording (Supporting Information: Figure S3) and an excellent pace mapping (≥11/12 leads), radiofrequency energy was delivered with a maximum power of 50 W and a target temperature of 55°C for the nonirrigated catheter, and 42°C for the irrigation catheter. Energy delivery continued for 60−180 s if there was an absence or reduction in PVC frequency within the first 30 seconds. Otherwise, the ablation was terminated and the originating foci were re‐evaluated.

Successful ablation was defined as follows: (1) absence of clinical PVCs for 30 minutes after the last radiofrequency ablation, both with and without isoproterenol stimulation; (2) absence of clinical PVCs during 12‐ to 24‐hour ECG monitoring following the ablation procedure. The originating foci of PVCs were determined based on the sites of successful ablation.

### 2.4 Statistical analysis

The Kolmogorov−Smirnov test was initially utilized to determine the distribution patterns of the continuous variables. Continuous variables with a normal distribution were presented as mean ± SD and compared suing the Student's *t* test to assess the differences between the groups. Alternatively, median (25th and 75th) was used to present the data, and the Mann−Whitney test was employed to calculate the differences between the groups. For categorical clinical variables, differences between groups were evaluated with the *χ* ^2^ or Fisher exact test when appropriate.

Propensity score‐matched (PSM) analysis was performed using the R MatchIt Package between PVC patients and healthy controls (HC) to mitigate the influence of different baseline characteristics on HRV and enhance the credibility of the results. Potential confounding factors including gender, age, and concomitant diseases were included in the analysis. The caliper width was set at 0.02, and the standardized mean difference was calculated to assess the balance of covariates after matching. The ratio of PVC group to HC group was 1:1.

Univariate and multivariate logistic regression analyses were conducted to investigate the potential predictors of the originating foci of PVCs. All variables with a significance level of <0.10 in the univariate logistic regression analysis were assessed for multicollinearity through collinearity diagnostics. Only variables with a variance inflation factor < 10 were included in the multivariate logistic regression analyses. Furthermore, receiver operating characteristics (ROC) curves were performed to examine the value of HRV variables in differentiating RVOT‐PVCs from LVOT‐PVCs. A *p* ≤ .05 was considered significant. All the statistical analyses were performed by the SPSS Statistics for Windows, v26.0 (SPSS Inc.), GraphPad Prism 8.0.2 (GraphPad) and the R software (Version 4.0.2; https://www.r-project.org).

## 3 RESULTS

### 3.1 Baseline characteristics of enrolled participants

The study ultimately included 106 patients with frequent OT‐PVCs and 106 healthy participants. Patients with frequent OT‐PVCs exhibited similar age (53.6 ± 14.0 vs 51.5 ± 11.2 years, *p* = .21) and a comparable proportion of males (48.1% vs 36.8%, *p* = .13) compared to the healthy participants. While the PVC group had a higher proportion of hypertension (40.6% vs. 24.5%, *p* = .01), there were similar comorbidities between the PVC and HC groups (Supporting Information: Table S1). More detailed baseline characteristics of patients with PVCs were shown in Table 1. All patients have normal cardiac geometry and systolic function. They also exhibited high PVC counts and burden, with β‐blockers being the most commonly prescribed pharmacological therapy in this population. Among the patients with OT‐PVCs, 53.8% showed a positive correlation between hourly PVC counts and the corresponding average heart rate, followed by a pattern of no correlation in 34.0% of cases (Supporting Information: Figure S2A−B).

**Table 1:** Baseline characteristics of enrolled patients with OT‐PVCs.

Variables	Overall (*n* = 106)	RVOT group (*n* = 73)	LVOT group (*n* = 33)	*p* Value
Age (years old)	53.6 ± 14.0	50.9 ± 14.0	59.8 ± 12.0	.002
Male (*n*, %)	51 (48.1)	36 (49.3)	15 (45.5)	.71
History duration (years)	1.7 ± 2.4	1.7 ± 2.6	1.6 ± 1.9	.86
Comorbidity				
Hypertension (*n*, %)	43 (40.6)	26 (35.6)	17 (51.5)	.12
Diabetes (*n*, %)	9 (8.5)	4 (5.5)	5 (15.2)	.13
CAD (*n*, %)	4 (3.8)	4 (5.5)	0 (0)	.31
Smoking (n, %)	26 (24.5)	18 (24.7)	8 (24.4)	.96
Previous treatment with AADs				
Class I AADs (*n*, %)	1 (0.9)	0 (0)	1 (3.0)	.31
Class Ⅱ AADs (*n*, %)	24 (22.6)	15 (20.5)	9 (27.3)	.44
Class Ⅲ AADs (*n*, %)	3 (2.8)	1 (1.4)	2 (6.1)	.23
Class Ⅳ AADs (*n*, %)	3 (2.8)	0 (0)	3 (8.3)	.03
Other AADs (*n*, %)	14 (13.2)	8 (11.0)	6 (18.2)	.36
Measurements on echocardiography				
LVEF (%)	65.0 (62.0−66.0)	65.0 (62.0−67.0)	64.0 (62.0−66.0)	.37
IVST (mm)	9.0 (9.0−10.3)	9.0 (8.5−10.0)	10.0 (9.0−11.0)	.40
Measurements on Holter				
PVC number (*n*)	19519 (13364−30612)	21128 (13621−30704)	16620 (12643−29957)	.46
Percentage of PVC (%)	18.0 (14.0−28.5)	19.0 (13.0−30.5)	17.0 (14.0−27.0)	.46
Mean heart rate (bpm)	74.3 ± 8.9	73.4 ± 8.5	76.2 ± 9.7	.13
PVC‐HR correlation				.2
Positive (*n*, %)	57 (53.8)	35 (47.9)	22 (66.7)	
Negative (*n*, %)	13 (12.3)	10 (13.7)	3 (9.1)	
No correlation (*n*, %)	36 (34.0)	28 (38.4)	8 (24.2)	

Abbreviations: AAD, antiarrhythmic drug; CAD, coronary artery disease; HR, heart rate; IVSTD, inter‐septum thickness; LVEF, left ventricular ejection fraction; LVOT, left ventricular outflow tract; OT‐PVC, outflow tract–premature ventricular complex; RVOT, right ventricular outflow tract.

### 3.2 Impaired HRV in patients with OT‐PVCs

**Figure 1:**
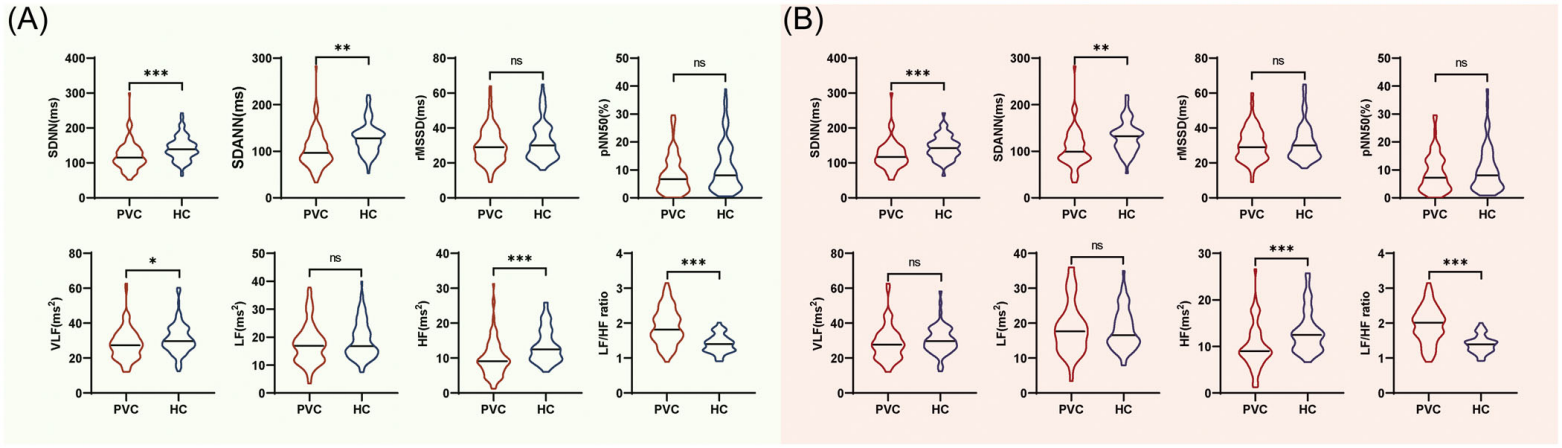
Comparison of HRV variables between PVC group and HC group before (A) and after propensity score‐matched analyses (B). *p < .05; **p < .01; ***p < .001. HC, healthy control; HF, high frequency; HRV, heart rate variability; PVC, premature ventricular complex; LF, low frequency; LVOT, left ventricular outflow tract; pNN50, percentage of normal RR intervals that differ by >50 millisecond. rMSSD, standard deviation of all NN intervals; RVOT, right ventricular outflow tract; SDANN, standard deviation of the average NN intervals; SDNN, standard deviation of all NN intervals; VLF, very low frequency.

The HRV was assessed in all participants using 24‐hour Holter recordings. In the frequency domain analyses, patients with OT‐PVCs exhibited decreased levels of HF (9.15 [6.51−12.21] vs. 12.47 [9.70−17.34] millisecond square, *p* < .001) and VLF (27.99 ± 8.87 vs. 31.05 ± 8.71 millisecond square, *p* < .05) compared to the healthy participants (Figure 1A). Although the levels of LF were comparable between the PVC and HC groups, the levels of LF/HF ratio were significantly higher in the PVC group (1.91 ± 0.51 vs. 1.41 ± 0.26, *p* < .001, Figure 1A). In the time domain analyses, significantly lower SDNN (115.00 [93.00−139.25] vs. 139.00 [120.00−159.25] millisecond, *p* < .001) and SDANN (96.50 [77.75−126.25] vs. 128.00 [104.00−143.00] millisecond, *p* < .001) were observed in the PVC group compared to the control group (Figure 1A). However, there was no significant difference in the levels of rMSSD and pNN50 between the PVC and HC groups (Figure 1A).

To account for the potential influence of baseline characteristics on HRV, PSM analyses were conducted to ensure result accuracy. A total of 118 participants (59 patients in the PVC group and 59 participants in the HC group) were included in the PSM analyses (Supporting Information: Table S1). After balancing the baseline characteristics, patients in the PVC group consistently exhibited higher levels of LF/HF ratio (2.01 [1.60−2.34] vs. 1.39 [1.22−1.51], *p* < .001) and lower levels of SDNN (117.00 [96.00−144.00] vs 142.00 [121.00−161.00] millisecond, *p* < .001), SDANN (99.00 [83.00−128.00] vs. 132.00 [104−146] millisecond, *p* = .001) and HF (9.02 [7.42−12.20] vs. 12.47 [9.41−14.81] millisecond square, *p* < .001) (Figure 1B).

### 3.3 Increased LH/HF ratios in patients with PVCs originating from RVOT

Due to the close proximity of the originating sites of RVOT‐PVCs and LVOT‐PVCs, they often exhibit similar characteristics on surface electrocardiograms, making it difficult to differentiate them. To further explore the relationship between HRV parameters and the originating foci of OT‐PVCs, patients with OT‐PVCs were divided into RVOT group (*n* = 73, 68.9%) and LVOT group (33, 31.1%), based on the identified originating foci of PVCs in the electrophysiological studies. Apart from a higher mean age in the LVOT group (59.8 ± 12.0 vs. 50.9 ± 14.0 years, *p* = .002), there were no significant difference in demographic characteristics, cardiac geometry and systolic function, PVC burden, and the correlation between hourly PVC counts and corresponding average heart rate (Table 1 and Supporting Information: Figure S2B).

**Figure 2:**
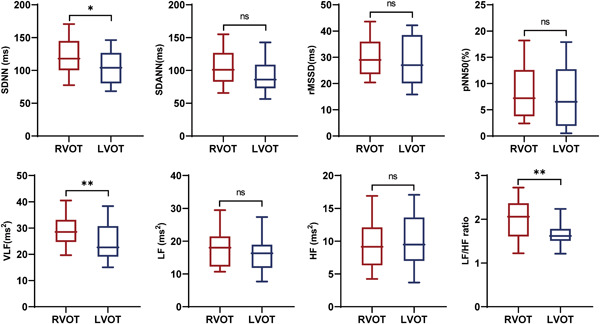
Comparison of HRV variables between RVOT and LVOT group. *p < .05; **p < .01. HF, high frequency; HRV, heart rate variability; pNN50, percentage of normal RR intervals that differ by >50 millisecond square; LF, low frequency; LVOT, left ventricular outflow tract; rMSSD, standard deviation of all NN intervals; RVOT, right ventricular outflow tract; SDANN, standard deviation of the average NN intervals; SDNN, standard deviation of all NN intervals; VLF, very low frequency.

Comparison of the HRV variables between the two groups revealed that patients in the RVOT group exhibited significantly higher levels of LF/HF ratio (2.04 ± 0.52 vs. 1.61 ± 0.36, *p* < .01), SDNN (118.00 [100.00−145.00] vs. 104.00 [80.00−127.00] millisecond, *p* < .05) and VLF (28.51 [23.64−33.18] vs. 23.44 [19.23−30.78] millisecond square, *p* < .05), but similar levels of other HRV variables (Figure 2). Univariate logistic regression analysis in patients with OT‐PVCs demonstrated that age (odds ratio [OR] 0.95, 95% confidence interval [CI] [0.91−0.98], *p* = .004), VLF (OR 1.06, 95% CI [1.00−1.12], *p* = .049), LF/HF ratio (OR 7.70, 95% CI [2.65–22.43], *p* < .001), and SDNN (OR 1.02, 95% CI [1.00–1.03], *p* = .03) were associated with RVOT‐PVCs. Multivariate logistic regression analysis demonstrated that only the LF/HF ratio (OR 5.74, 95% CI [1.88–17.55], *p* = .002) independently correlated with RVOT‐PVCs (Table 2).

**Table 2:** Univariate and multivariate logistic regression analyses.

Variables	Odds ratio	95% CI	*p* Value
*Univariate analysis*			
Age	0.95	0.91–0.98	.004
VLF	1.06	1.00–1.12	.049
LF/HF ratio	7.70	2.65–22.43	<.001
SDNN	1.02	1.00–1.03	.03
*Multivariate analysis*			
LF/HF ratio	5.74	1.88–17.55	.002

Abbreviations: CI, confidence interval; HF, high frequency; LF, low frequency; SDNN, standard deviation of all NN intervals; VLF, very low frequency.

### 3.4 The value of the LF/HF ratio in differentiating between RVOT‐PVCs and LVOT‐PVCs

**Figure 3:**
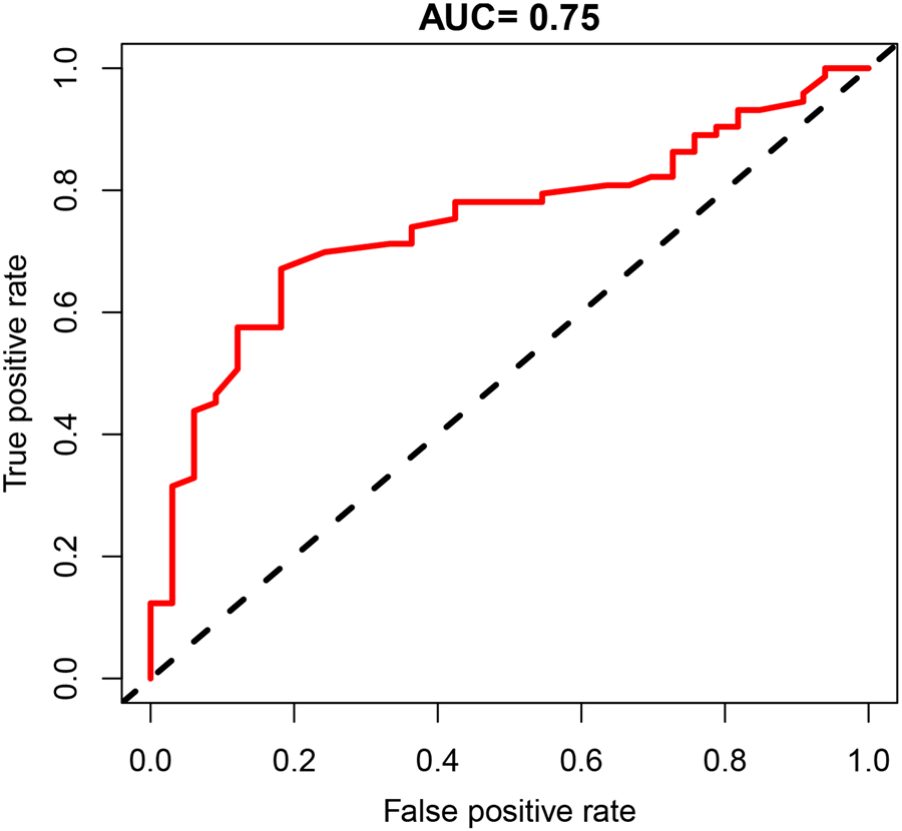
ROC curve to investigate the value of the LF/HF ratio in distinguishing between RVOT‐PVCs and LVOT‐PVCs. AUC, the area under the curve; HF, high frequency; LF, low frequency; LVOT, left ventricular outflow tract; PVC, premature ventricular complex; ROC, receiver operating characteristics; RVOT, right ventricular outflow tract.

ROC curve analysis was conducted to assess the effectiveness of the LF/HF ratio in differentiating between RVOT‐PVCs and LVOT‐PVCs. ROC curve demonstrated that the LF/HF ratio significantly distinguished patients with RVOT‐PVCs from those with LVOT‐PVCs (the area under the curve [AUC] = 0.75, 95% CI 0.65−0.84, *p* < .001). The optimal cutoff value for the LF/HF ratio in discriminating RVOT‐PVCs was determined to be 1.795, based on achieving the best balance between sensitivity and specificity. An LF/HF ratio ≥ 1.795 predicted RVOT‐PVCs with a sensitivity of 67.1% and a specificity of 81.9%. The positive predictive value and negative predictive value for RVOT‐PVCs were found to be 89.1% and 52.9%, respectively (Figure 3).

## 4 DISCUSSION

The primary finding of our study revealed that impaired HRV was not only associated with OT‐PVCs but, more importantly, correlated with the specific originating foci of OT‐PVCs. The key findings of our study can be summarized as follows: (1) patients with frequent OT‐PVCs exhibited decreased levels of HF, SDNN, and SDANN, but increased levels of the LF/HF ratio compared to participants in the control group; (2) patients with RVOT‐PVCs demonstrated higher levels of the LF/HF ratio, SDNN, and VLF than patients with LVOT‐PVCs, while maintaining similar levels of other HRV variables; (3) the LF/HF ratio independently correlated with RVOT‐PVCs and possessed clinical significance in effectively distinguishing between RVOT‐PVCs and LVOT‐PVCs.

The cardiac ANS plays a crucial role in the initiation of OT‐VAs. In proximity to the aortic root and RVOT, there exist ganglia plexus comprised of sympathetic and parasympathetic neurons and fibers.^15^ Animal and human experiments demonstrated that stimulating the sympathetic fibers at the RVOT could induce VAs.^16^ Human studies revealed that enhancing cardiac sympathetic activity resulted in a shortened RVOT activation recovery interval and increased repolarization dispersion.^17^ Activation of β‐adrenergic receptors on the myocardial surface in the RVOT can induce premature ventricular contractions (PVCs) via cyclic adenosine monophosphate (cAMP)‐mediated delayed after depolarization.^18^ Conversely, stimulation of parasympathetic nerves can inhibit triggered activity by activating Gi protein through cardiac muscarinic receptors, thereby counteracting the increased cAMP production induced by catecholamines.^19^ The balance between sympathetic and parasympathetic activity is pivotal for maintaining cardiac electrophysiological stability in the ventricular outflow tract. Disruption of this balance, characterized by increased sympathetic activity and/or decreased parasympathetic activity, can lead to OT‐PVCs via the cAMP‐mediated pathway. Previous studies on patients with OT‐VAs also indicated alterations in cardiac ANS function before the onset of VAs.^20^, ^21^ Our study further demonstrated that patients with frequent OT‐PVCs exhibited impaired HRV, indicative of cardiac ANS dysfunction in these individuals. On the contrary, it is plausible that the impaired HRV may be partially attributed to the observed PVCs. The decrease in arterial pressure caused by PVCs could trigger the activation of cardiac sympathetic nerves through baroreceptor reflex, aiming to restore normal arterial pressure. This response could be exacerbated by impaired parasympathetic function.^22^, ^23^, ^24^ Consequently, this might explain the relatively low efficacy of β‐receptor blockers in certain patients with PVCs.^25^

Another significant finding from our study was that patients with RVOT‐PVCs exhibited higher levels of LF/HF ratio compared to patients with LVOT‐PVCs. This discovery indicates that patients with RVOT‐PVCs may have more severe cardiac ANS dysfunction. While both the RVOT and the aortic root contain ample ganglia plexus,^15^ the role of cardiac ANS in the pathogenesis of RVOT‐PVCs appears to be more prominent. Animal and human studies demonstrated that high‐frequency stimulation at pulmonary artery or left stellate ganglion could induce RVOT‐PVCs. These inductions were prevented by pulmonary artery denervation or use of adrenergic receptor antagonists.^16^, ^26^, ^27^, ^28^ However, there are few studies investigating the role of cardiac ANS in LVOT‐PVCs. Additionally, the RVOT contains more myocardium compared to the aortic root, resulting in a higher level of coupling between myocardium and cardiac ANS fibers in the RVOT region. The differences in the coupling of myocardial and cardiac ANS fibers may attribute to variations in the LF/HF ratios between RVOT‐PVCs and LVOT‐PVCs. Previous studies implied that RVOT‐PVCs might have more detrimental effects on hemodynamics and cardiac remodeling compared to LVOT‐PVCs,^29^ which might promote more significant remodeling of the cardiac ANS. The findings of our study revealed similar levels of HF and LF between patients with RVOT‐ and LVOT‐PVCs. This outcome suggests that cardiac ANS imbalance, rather than the sympathetic or parasympathetic nerves specially, is independently associated with the origin of OT‐PVCs. Earlier studies emphasized the significance of cardiac sympathetic nerves on ventricular cardiomyocytes, but a recent study has also highlighted the crucial role of cardiac parasympathetic nerves in ventricles.^30^ Thus, our study underscores the importance of cardiac ANS imbalance in the pathophysiology of RVOT‐PVCs.

The observation of distinct LF/HF ratio levels between patients with RVOT‐PVCs and LVOT‐PVCs holds clinical significance for the management of patients with OT‐PVCs. Differentiating between RVOT‐PVCs and LVOT‐PVCs before RFCA is crucial in planning procedures to ensure accurate mapping and ablation. Several surface ECG criteria and algorithms exist for distinguishing between RVOT‐PVCs and LVOT‐PVCs, primarily based on the anatomical location of the origin and the conduction pathway of the PVCs.^31^, ^32^ However, the complex anatomy of the outflow tract, overlapping common sites of PVC origin, and potential factors such as cardiac rotation, lead positions, and preferential conduction of PVCs may limit the efficacy of typical surface ECG criteria.^31^, ^33^ Our study demonstrated significant clinical value of the LF/HF ratio in identifying the originating foci of OT‐PVCs, which was independent of cardiac anatomy. Therefore, the LF/HF ratio may provide additional value to existing ECG criteria and algorithms in the differentiation between RVOT‐PVCs and LVOT‐PVCs.

### 4.1 Study limitation

There are several limitations to our study. Firstly, the relatively small sample size of patients used to investigate the value of the LF/HF ratio in distinguishing between RVOT‐PVCs and LVOT‐PVCs may impact the reliability of the conclusions. Additionally, although the LF/HF ratio may offer additional value to current ECG criteria and algorithms in differentiating between RVOT‐PVCs and LVOT‐PVCs, our study did not collect surface ECG data to investigate this aspect. Therefore, further prospective studies are required to explore the clinical significance of HRV variables in patients with OT‐PVCs. Lastly, the advantages of high‐density mapping in patients with OT‐PVCs has been validated in previous studies.^34^, ^35^ However, high‐density mapping is not routinely used in our center, and no patients in this study received high‐density mapping.

## 5 CONCLUSION

Patients with frequent outflow tract PVCs exhibited impaired HRV. Additionally, the LF/HF ratio played a significant role in determining the origin of outflow tract PVCs.

## CONFLICT OF INTEREST STATEMENT

The authors declare no conflict of interest.

## Supporting information

Supporting information.

## Data Availability

The data used during the study are available from the corresponding author on reasonable request
