# Supplementary material for: The significance of heart rate variability in patients with frequent premature ventricular complex originating from the ventricular outflow tract
Source: Clin Cardiol. 2023 Oct 19;47(1):e24174. doi: 10.1002/clc.24174 (PMC10766131; doi:10.1002/clc.24174)
Supplement: Supplementary file 1 — Supporting information. [file CLC-47-e24174-s001.docx]

**Supporting information**

**The significance of heart rate variability in patients with frequent premature ventricular complex originating from the ventricular outflow tract**

Baowei Zhang, MD^1†^, Jinbo Yu, MD^1†^, Yizhang Wu, MD^1^, Xiaorong Li, MD^1^, Xin Xie, MD^1^, Aibin Tao, MD^2*^, Bing Yang, MD^1*^

Correspondence to: Bing Yang, bingyang@tongji.edu.cn

This file contains: Figures and figure legends (pages 2-4)

Table (pages 5)

**Figure S1**


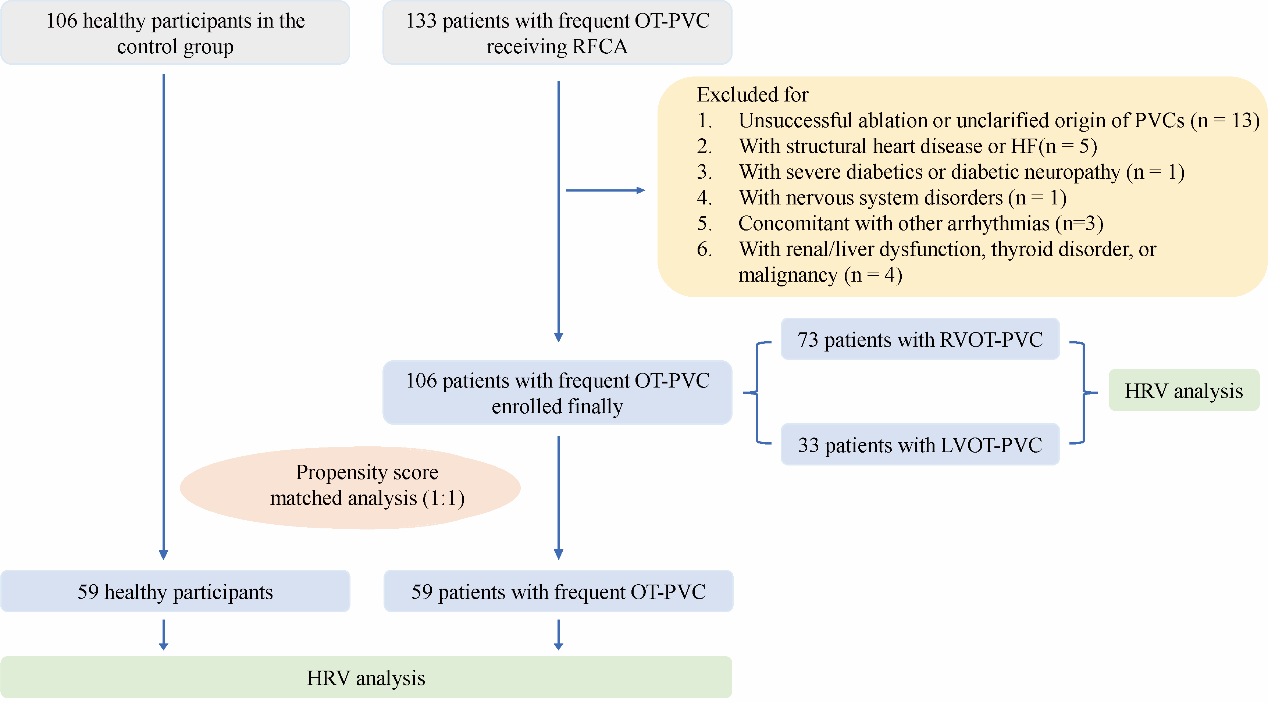


**Figure S1.** Participant selection and data analysis flowchart

OT-PVC, outflow tract premature ventricular complex; HRV, heart rate variability; RFCA, radiofrequency catheter ablation; RVOT, right ventricular outflow tract; LVOT, left ventricular outflow tract.

**Figure S2**


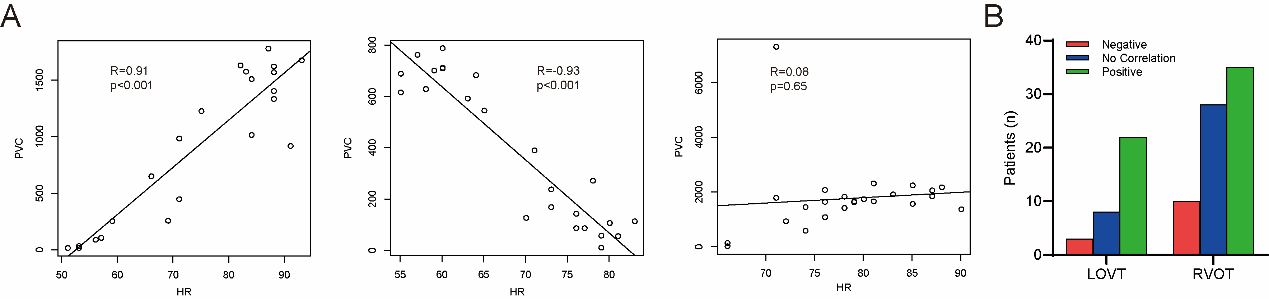


**Figure S2.** Comparison of distribution patterns between RVOT-PVC and LVOT-PVC groups

A. Correlation between hourly heart beat and hourly PVC counts.

B. Both RVOT-PVC and LVOT-PVC groups had similar correlation between hourly heart beat and hourly PVC counts.

**Figure S3**


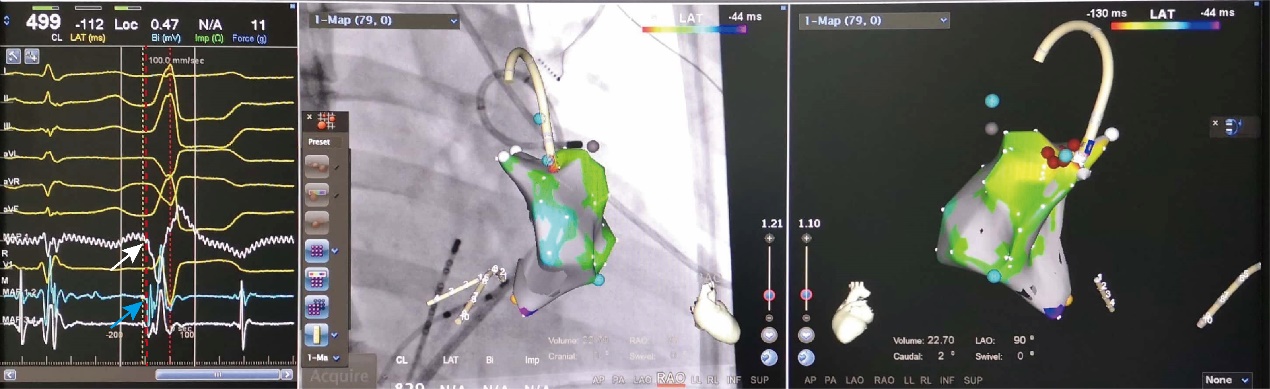


**Figure S3.** Representative image of bipolar electrogram (light blue arrow in the left figure), unipolar electrogram (white arrow in the left figure), and origin of the PVCs on fluoroscopy and 3-dimensional mapping in a patient with RVOT-PVCs.

**Table S1. Baseline characteristics of participants in PVC group and HC groups before and after propensity score matching**

|  | Unmatched (n = 212) | | | | PSM 1:1 (n = 118) | | | |
| --- | --- | --- | --- | --- | --- | --- | --- | --- |
|  | PVC group  (n=106) | HC group  (n=106) | SMD | p | PVC group  (n=59) | HC group  (n=59) | SMD | p |
| Age  (years) | 53.6 ± 14.0 | 51.5 ± 11.2 | 0.173 | 0.21 | 50.7 ± 13.7 | 52.4 ± 11.6 | 0.139 | 0.45 |
| Male  (n, %) | 51 (48.1) | 39 (36.8) | 0.231 | 0.13 | 21 (35.6) | 21 (35.6) | <0.001 | 1.00 |
| Hypertension (n, %) | 43 (40.6) | 26 (24.5) | 0.347 | 0.02 | 16 (27.1) | 16 (27.1) | <0.001 | 1.00 |
| Diabetes (n, %) | 9 (8.5) | 7 (6.6) | 0.071 | 0.80 | 1 (1.7) | 1 (1.7) | <0.001 | 1.00 |
| CAD  (n, %) | 4 (3.8) | 0 (0.0) | 0.280 | 0.13 | 0 (0) | 0 (0) | <0.001 | NA |
| Smoking (n, %) | 26 (24.5) | 17 (16.0) | 0.212 | 0.17 | 10 (16.9) | 12 (20.3) | 0.087 | 0.81 |

PVC, premature ventricular complex; HC, healthy control; PSM, propensity score matching; SMD, standardized mean difference; CAD, coronary artery disease.
